# Investigational Monoclonal Antibodies in the Treatment of Multiple Myeloma: A Systematic Review of Agents under Clinical Development

**DOI:** 10.3390/antib8020034

**Published:** 2019-05-24

**Authors:** Ahmad Iftikhar, Hamza Hassan, Nimra Iftikhar, Adeela Mushtaq, Atif Sohail, Nathaniel Rosko, Rajshekhar Chakraborty, Faryal Razzaq, Sonia Sandeep, Jason Neil Valent, Abraham Sebastian Kanate, Faiz Anwer

**Affiliations:** 1 Department of Internal Medicine, The University of Arizona, Tucson, AZ 85721, USA; ahmad.iftikhar167@gmail.com; 2 Department of Internal Medicine, Rochester General Hospital, Rochester, NY 14621, USA; Hamza.Hassan@rochesterregional.org (H.H.); atif.sohailmd@gmail.com (A.S.); 3 Dow University of Health Sciences, Karachi 74200, Pakistan; nimra.b@gmail.com; 4 Department of Internal Medicine, University of Pittsburgh Medical Center, McKeesport, PA 16148, USA; adeela_mushtaq@hotmail.com; 5 Taussig Cancer Center, Cleveland Clinic, Cleveland, OH 44106, USA; roskon@ccf.org (N.R.); chakrar2@ccf.org (R.C.); valentj3@ccf.org (J.N.V.); 6 Foundation University Medical College, Islamabad 44000, Pakistan; frazzaq90@gmail.com; 7 Department of Pathology, Wilson Medical Center, Wilson, NC 27893, USA; soniasandeep123@gmail.com; 8 Department of Hematology Oncology, West Virginia University, Morgantown, WV 26506, USA; ASKANATE@hsc.wvu.edu

**Keywords:** Multiple myeloma, immunotherapy, antibody, targeted therapy, molecular targets, bispecific antibodies, immune checkpoint inhibitors, Antibody Drug Conjugate

## Abstract

Background: Immunotherapy for multiple myeloma (MM) has been the focus in recent years due to its myeloma-specific immune responses. We reviewed the literature on non-Food and Drug Administration (FDA) approved monoclonal antibodies (mAbs) to highlight future perspectives. We searched PubMed, EMBASE, Web of Science, Cochrane Library and ClinicalTrials.gov to include phase I/II clinical trials. Data from 39 studies (1906 patients) were included. Of all the agents, Isatuximab (Isa, anti-CD38) and F50067 (anti-CXCR4) were the only mAbs to produce encouraging results as monotherapy with overall response rates (ORRs) of 66.7% and 32% respectively. Isa showed activity when used in combination with lenalidomide (Len) and dexamethasone (Dex), producing a clinical benefit rate (CBR) of 83%. Additionally, Isa used in combination with pomalidomide (Pom) and Dex resulted in a CBR of 73%. Indatuximab Ravtansine (anti-CD138 antibody-drug conjugate) produced an ORR of 78% and 79% when used in combination with Len-Dex and Pom-Dex, respectively. Conclusions: Combination therapy using mAbs such as indatuximab, pembrolizumab, lorvotuzumab, siltuximab or dacetuzumab with chemotherapy agents produced better outcomes as compared to monotherapies. Further clinical trials investigating mAbs targeting CD38 used in combination therapy are warranted.

## 1. Introduction

Multiple myeloma (MM) remains an incurable hematologic malignancy. The American Cancer Society estimates 32,110 new cases of MM in the year 2019 with estimated deaths about 12,960 [1]. MM is a multi-clonal disease characterized by significant intra-tumor heterogeneity, which in turn leads to clonal evolution and tiding along the trajectory of disease [2,3,4]. Despite ongoing efforts to advance MM precision and personalized therapy using next generation sequencing (NGS), whole-genome sequencing (WGS), RNA sequencing (RNA-Seq) and sequencing panels for direct targeting, the field is still in its infancy and its broad clinical applicability remains undetermined. The key treatment strategies currently utilized in contemporary practice consist of three-drug regimens, typically including a proteasome inhibitor (PI), immunomodulatory drug (IMiD) or a mAb, along with dexamethasone. Despite many therapeutic advances, MM patients invariably relapse after a period of remission mainly due to development of resistance to therapy, associated clonal proliferation, and/or sub clonal divergence [5].

Monoclonal antibodies provide an important scaffold for signaling mechanisms, which we classified as follows. (1) Complement dependent cytotoxicity (CDC): The antibodies bind to the tumor cell surface, which stimulates proteolytic enzymes to form a membrane attack complex to kill tumor cells by lysing the cell membrane, [6], (2) Antibody-dependent cellular cytotoxicity (ADCC): mAbs bind to tumor cells by interaction between Fc region of an antibody and the Fc receptor on immune cells such as neutrophils, natural killer (NK) cells and macrophages. This subsequent interaction leads to phagocytosis of mAb-tumor cell conjugates with resultant cell lysis by NK cells, [7], (3) mAbs can function as agonists to activate apoptosis, (4) mAbs function as antagonists to block intracellular signaling pathways to stop cell proliferation, (5) Antibody-drug conjugate (ADC) can act as a carrier to transport cytotoxic agents inside tumor cells to cause tumor cell lysis decreasing systemic toxicities [8]. These anti-tumor mechanisms can be achieved by aiming a target (receptor/ligand/chemokine/cytokine) that is either present in the cancer-milieu, trans-membrane or intracellularly.

Monoclonal antibodies have produced favorable results as combination therapy with PIs and IMiDs. Currently, daratumumab (Dara) is Food and Drug Administration (FDA)-approved as monotherapy [9] and combination therapy along with elotuzumab (Elo) for the relapsed and refractory MM (RRMM). Combination therapy trials for RRMM showed improved efficacy for the mAb containing arms such as POLLUX, CASTOR, ELOQUENT-2 and ELOQUENT-3 trials assessing Dara and Elo, respectively, and reported ORR 93% vs. 76% (Dara-Rd vs. Rd), 83% vs. 63% (Dara-Vd vs. Vd), 79% vs. 66% (Elo-Rd vs. Rd) and 53% vs. 26% (Elo+Pom+Dex vs Pom+Dex) [10,11,12,13]. For newly diagnosed multiple myeloma (NDMM), a multi-center phase III (ALCYONE) trial using daratumumab in a four drug combination of bortezomib, melphalan and prednisone (VMp) showed 18-month PFS of 71.6% for Dara-VMp versus 50.2% for VMp alone [14].

The goal of mAb combination regimens is to deepen initial responses with minimal residual disease-negative status which predicts improved PFS and overall survival (OS) [15]. Unlike many currently approved non-targeted therapies for MM, targeted mAbs help overcome myeloma-associated immune dysregulation [16], synergistically enhance immunomodulation and have the potential to improve immune surveillance. Immune therapies for MM include the use of mAbs targeting the surface or non-surface receptors with naked or linked antibodies, cancer vaccines and adaptive immunotherapy utilizing genetically engineered T-cells (CAR-T) [17].

We reviewed the literature on investigational non-FDA approved mAbs and outlined their pathogenic targets, trial data, reported adverse events and have included a discussion of future perspectives in this drug category.

## 2. Methods

### 2.1. Search Strategy

We searched PubMed, EMBASE, Web of Science, Cochrane Library and ClinicalTrials.gov on 09/27/2018. While running our search, we applied the last 10 years’ filter and excluded non-human studies. We also reviewed bibliographies of review articles to include additional relevant studies.

### 2.2. Eligibility Criteria

This review includes Phase I/II clinical trials of mAbs utilized in the treatment of MM, which were either complete or currently recruiting. We included studies performed in the last ten years (2008–2018) with reported efficacy outcomes. We excluded studies and clinical trials on Dara and Elo as they are already FDA approved for the treatment of RRMM. We excluded review articles and trials with no reported efficacy outcomes.

### 2.3. Study Selection and Data Extraction

We screened articles based on titles/abstracts first and later by reading full texts of eligible articles by two reviewers independently (HH/NI). We used a data extraction form in Excel to extract data such as author, study design, year, the total number of patients, target receptor, antibody, antibody drug regimen, median prior lines of therapy, dosage, number of dosage cycles, efficacy outcomes and adverse outcomes. For most of the studies, the primary outcome extracted was overall response rate (ORR) and overall response (OR). With respect to clinical outcomes, we collected complete response (CR), stable disease (SD), partial response (PR), progressive disease (PD), overall survival (OS) and progression-free survival (PFS) rates. Regarding toxicities and adverse outcomes, we extracted data about common adverse events and > grade III toxicities.

## 3. Results

**Figure 1:**
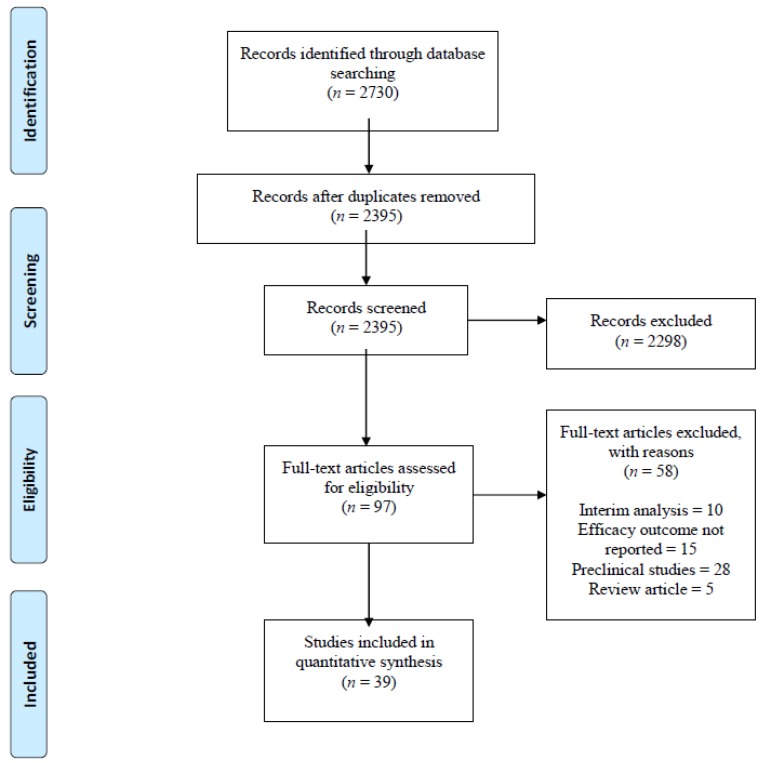
PRISMA flow diagram. Records identified through PubMed, Embase, Cochrane, SCOPUS and Clinical.Trials.gov database searches.

We identified 2730 studies using PubMed (90), Cochrane (150), EMBASE (725), SCOPUS (1749) and ClinicalTrials.gov (16). After excluding 335 duplicates, we read titles and abstracts of the remaining 2395 articles and excluded an additional 2298 articles that were review articles, expert opinions, preclinical studies, monoclonal antibodies targeting cancers other than MM and studies on daratumumab and Elotuzumab. We read full-length manuscripts of the remaining 97 articles and excluded 58 articles based on the following reasons: no efficacy outcomes reported (15), interim analysis (10), preclinical studies (28), review articles (5). Thirty-Nine (39) clinical trials that met our inclusion criteria included 1906 patients. (Figure 1: PRISMA flow diagram).

We evaluated the following surface receptor antibodies (n denotes the number of studies): Dacetuzumab (anti-CD40, *n* = 2), Lucatumumab (anti-CD40, *n* = 1), Isatuximab (anti-CD38, *n* = 6), MOR202 (anti-CD38, *n* = 1), IPH 2101 (anti-KIR, *n* = 2), Milatuzumab (anti CD74, *n* = 1), BI-505 (anti-ICAM1, *n* = 1), Figitumumab (anti-IGF1, *n* = 1), AVE 1642 (anti-IGF1, *n* = 1), PAT SM6 (anti-GRP-78, *n* = 1). (Table 1).

We also evaluated the following Non-surface receptor antibodies: Bevacuzumab (anti-VEGF, *n* = 2), Siltuximab (anti-IL6, *n* = 5), Atacicept (anti-BAFF, *n* = 1), Tabalumab (anti-BAFF, *n* = 2), Nivolumab (anti-PD-1, *n* = 2), Pembrolizumab (anti-PD-1, *n* = 2), Pidilizumab (anti-PD-1, *n* = 1), F50067 (anti-CXR4, *n* = 1), Napatumumab (anti-TRAILR, *n* = 1), Lorvotuzumab (anti-CD56, *n* = 2), Indatuximab Ravtansine (anti-CD138 antibody drug conjugate, *n* = 3) (Table 2).

We summarized adverse effects related to mAb drugs in Table 3.

### 3.1. Surface Receptor-Targeting Antibodies

#### 3.1.1. CD38

CD38 is highly expressed in MM cells. It plays a key role in the growth of cells and their survival. CD38 acts as a surface receptor to regulate the proliferation of T-cells by means of cytokine recruitment and cell adhesion. It also acts as an ectoenzyme in the synthesis of cyclic adenosine diphosphate ribose (cADPR) and the hydrolysis of cADPR to ADPR and generates NAADP from NADP. NAADP, cADPR and ADPR play important roles in the regulation of calcium influx and other cellular signaling pathways [18,19]. Isatuximab is anti-CD38 mAb that produces its anticancer effects by means of CDC, ADCC or direct toxicity. Isatuximab as monotherapy achieved an ORR of 32% and 24% in patients who had failed 6 and 5 lines of therapy, respectively [20,21]. When isatuximab was combined with lenalidomide and dexamethasone in doses of 3, 5 or 10mg/kg, it produced an ORR of 64.5% and 56% in patients with 6 and 5 prior lines of therapies respectively [22,23]. The use of isatuximab + Rd in doses of 10mg/kg and 20mg/kg achieved a similar ORR of 50% with a clinical benefit response (CBR) significantly higher at 83% with a 10mg/kg dose as compared with CBR of 50% with a 20mg/kg dose [24]. Mikhael et al. studied isatuximab in combination with pomalidomide and dexamethasone in patients with 4 prior lines of therapy and achieved the highest PR of 62%. In that study, 1 patient achieved CR and 8 patients achieved very good partial response (VGPR) with clinical benefit response (CBR) of 73% [25]. Patients tolerated Isatuximab at 10mg/kg dosing while 20mg/kg caused adverse events such as pneumonia, fatigue, hypokalemia, anaphylaxis and febrile neutropenia. A randomized phase III trial to compare isatuximab + Pd versus Pd is currently ongoing [26]. MOR202 is another anti-CD38 antibody under investigation as a single agent and in combination with lenalidomide and pomalidomide separately. Investigators saw long lasting tumor control with MOR202 monotherapy with patients achieving 19% PR and 13% VGPR, MOR202 + lenalidomide resulted in 5 out of 7 patients achieving PR and MOR202 + pomalidomide resulted in achieving CR in 2 out of 5 patients [27].

#### 3.1.2. CD40

CD40 is a surface receptor that is highly expressed on cells with increased potential for proliferation. It has been detected in various B-cell neoplasms including MM [28]. Dacetuzumab is an anti-CD40 mAb that is being tested both as single and combination therapy. The use of Dacetuzumab as monotherapy in 44 RRMM patients with prior 5 lines of therapy achieved no ORR with 20% patients in SD [29]. The use of Dacetuzumab + Rd in 36 RRMM with prior 4 lines of therapy produced OR of 39% with PR of 33% [30]. Lucatumumab is another anti-CD40 mAb with two distinct anti-tumor activities; it stops CD40-CD40L dependent cell growth and causes tumor cell lysis by ADCC [31]. The use of Lucatumumab as monotherapy in 28 patients who relapsed to 3 lines of therapy achieved PR in one patient and 43% of patients achieved SD [31].

#### 3.1.3. KIR

Killer-cell immunoglobulin like receptors (KIR) are transmembrane glycoproteins that express on the surface of nature killer (NK) cells. KIRs are mostly inhibitory as they decrease the cytotoxic potential of NK cells. NK-cells play an important role in protection against cancer development. IPH-2101 is an anti-KIR mAb that enhances the cytotoxic potential of NK cells against cancer cells by ADCC. IPH-2101 did not produce any ORR when used as monotherapy in RRMM [32]. Even in phase I-II trials, IPH-2101 used as monotherapy in smoldering MM did not achieve a clinical response [33]. When IPH-2101 was used in combination therapy with lenalidomide in the phase-I trial, ORR of 33.3% (n = 5) was achieved with two VGPR and three PR while six patients had SD. IPH-2101 in a dose range of 0.2–2mg/kg was also well tolerated [34].

#### 3.1.4. CD74

CD74 is a membrane glycoprotein found on B-cells, MM cells and monocytes. Because of its rapid internalization, association with major histocompatibility complex class II and restricted expression by normal cells, CD74 can deliver cytotoxic drugs inside cancer cells [35]. These properties led to a phase I clinical trial of anti-CD74 humanized mAb, milatuzumab for treating RRMM patients. 25 MM patients who relapsed after 5 prior lines of therapies were treated with milatuzumab and no ORR was achieved. Out of 19 patients who completed treatment, five had SD for 3 months after therapy while one had SD for 17 months. CRS was seen in five patients [36]. There is a need for further trials using milatuzumab with combination chemotherapy.

#### 3.1.5. ICAM-1 (CD54)

Intercellular adhesion molecule (ICAM-1) helps in the adhesion of MM cells to marrow stromal cells with resultant tumor proliferation. The ICAM-1 overexpression is associated with resistance to chemotherapy resulting in advanced disease [37,38]. Targeting ICAM-1 by humanized IgG1 mAb BI-505 as monotherapy in 35 RRMM patients did not achieve an ORR with 24% patients having SD and 65% having PD [39].

#### 3.1.6. Insulin Like Growth Factor-1 (IGF-1)

IGF-1 receptor and binding proteins play a critical part in MM pathogenesis by up regulating proliferation, angiogenesis, tumor survival and osteolysis by stimulating osteoclast activity. IGF-1 attracts MM cells to bone marrow, up regulates expression of anti-apoptotic molecules and down regulates pro-apoptotic molecules hence stimulating proliferation of MM cells. The expression of IGF-1 in MM is associated with poor outcomes and causes resistance to therapy [40]. In phase I trials, investigators have tested AVE1642 and figitumumab (Anti-IGF-1R mAb) as monotherapy versus combination with bortezomib or dexamethasone. The use of figitumumab as monotherapy achieved no ORR while its combination with dexamethasone achieved PR in 6 out of 27 patients [41]. When AVE1642 was used as monotherapy, it produced MR in 1 patient while maintaining SD in 7 out of 17 patients. AVE1642 + dexamethasone produced CR and PD in one patient each while SD in 3 out of 11 patients [42]. Despite favorable safety as both monotherapy and in combination, investigators did not consider the response as significant.

#### 3.1.7. GRP78

Glucose regulated protein 78 (GRP78) is a cellular protein that is found mostly in the endoplasmic reticulum (ER) and mitochondria and helps in protein assembly and controls ER stress signaling. GRP78 is an anti-apoptotic protein and can translocate to cytosol and the cell surface in tumor cells where it plays a role in angiogenesis, tumor progression and metastasis. It also results in resistance to proteasome and BRAF inhibitors [43]. PAT-SM6 targets GRP-78 and causes cytotoxicity in MM cells by inducing apoptosis and CDC [44]. Although PAT-SM6 was well tolerated in a phase I trial as a single agent in 12 RRMM patients with 4 lines of therapy it failed to produce OR with SD in 33.3% [44]. The favorable safety profile makes it a candidate for further trials with combination therapies [45].

### 3.2. Non-Surface Receptor Targeting Antibodies

#### 3.2.1. CXCR-4

CXCR-4 is expressed in many hematological malignancies and is thought to have a role in cancer cell survival [46]. CXCR-4/SDF1 axis plays an important role in the localization of MM cells in bone marrow, regulation of MM cells trafficking by adhesion, invasion and mobilization of MM cells out of the bone marrow [47]. F50067 is a humanized IgG1 anti-CXCR-4 mAb and exerts its ant-tumor effects by decreasing the interaction of MM cells with bone marrow microenvironment and resultant toxicity through ADCC and CDC. Fouquet et al studied 14 RRMM patients as F50067 monotherapy and combination of F50067 + Rd (low-dose dexamethasone) with 66.7% ORR (≥PR) in combination cohort and OR of 33.3% (≥SD). Investigators discontinued the study due to hematological toxicities [48].

#### 3.2.2. Interleukin 6 (IL 6)

IL-6 is a cytokine that mediates B- and T-cell immune function. It is an important regulator of the inflammatory response and mediates differentiation and proliferation of plasma cells. Siltuximab is an anti-IL6 mAb that demonstrated potent activity against MM in preclinical studies in combination with bortezomib and dexamethasone [49,50].

The Brighton et al. phase II randomized control trial (RCT) of siltuximab monotherapy versus placebo to evaluates whether siltuximab can delay the progression of high risk SMM to MM. One year PFS was 84.5% in siltuximab group versus 74.4% in the placebo group. This trial failed to meet the hypothesis that siltuximab can increase PFS by 14% [51]. Orlowski et al. did a phase II RCT of comparison of siltuximab + bortezomib versus bortezomib monotherapy. They demonstrated no significant difference in mean progression free survival (mPFS) (8 vs. 7.6 months), mOS (20.8 vs. 26.8 months), CR (11 vs. 7%) and ORR of 55 versus 47% [52]. In another trial of siltuximab as monotherapy versus siltuximab and dexamethasone in patients who failed four previous lines of therapy, siltuximab monotherapy did not achieve OR with SD of 62% and PD of 39%. In combination therapy, ORR of 23% was achieved with PFS of 3.7 months, SD 57% and PD 17% [53]. Suzuki et al. evaluated siltuximab + Rd in 9 patients with 1-2 previous lines of therapy and reported CR in 22% and PR in 44% [54].

#### 3.2.3. Vascular Endothelial Growth Factor (VEGF)

Angiogenesis plays a vital role in the growth and spread of neoplasms. MM can secrete VEGF, resulting in an increase in bone marrow micro-vascularity, which is an important prognostic factor in MM [55,56,57]. VEGF stimulates the endothelium of microvasculature and bone marrow stromal cells to secrete IL-6, which is also a strong growth factor for malignant MM cells [58]. Bevacizumab is a mAb, which binds VEGF and blocks its effects on angiogenesis and proliferation [59]. Callender et al. evaluated the role of bevacizumab + Rd in 31 patients who had failed prior median 3 lines of therapy and described OR of 70%. The results were not considered superior to Rd alone where OR was 60% [60]. In an RCT by Somlo et al. using bevacizumab alone versus bevacizumab ± thalidomide in 6 patients each with prior median three lines of therapy, bevacizumab monotherapy did not achieve OR while the addition of thalidomide slightly improved the response with PR of 33% [61]. In another RCT by White et al. for comparing bevacizumab + bortezomib in 49 patients versus bortezomib + placebo in 53 patients with 1-3 previous lines of therapy, there was no statistically significant difference in ORR (51% vs. 43.3%) [62].

#### 3.2.4. B-Cell Activating Factor (BAFF)

BAFF and APRIL (a proliferation-inducing ligand) are members of the TNF-α family and are highly expressed in MM. Both targets activate phosphatidylinositol-3 (PI-3) kinase and mitogen activated protein kinase with resultant up regulation of MCL-1 and BCL-2 anti-apoptotic proteins in MM. They also protect MM cells from dexamethasone-induced apoptosis [63]. Their levels are also associated with disease severity [64]. Atacicept and tabalumab are anti-BAFF mAbs. Atacicept blocks soluble forms of BAFF and APRIL, while tabalumab blocks both soluble and membrane bound forms of BAFF [65]. Rossi et al. evaluated atacicept as monotherapy in 12 patients who had failed 5 prior lines of therapy and no OR was achieved [65]. Shinsuki et al. studied tabalumab in doses of 100-200 mg in combination with Vd in RRMM patients with 1-5 prior lines of therapy and reported a combined OR of 56.3% and tabalumab was well tolerated [66]. Raje et al. studied tabalumab 100mg, 300mg and placebo + Vd each and found no statistically significant difference between OR and PFS. They reported an ORR of 58.1%, 59.5% and 61.1% with tabalumab of 100mg, 300mg and placebo + Vd respectively [67]. RRMM patients who had low BAFF levels achieved a moderately long PFS, which points to its role as an important prognostic indicator in RRMM [67].

#### 3.2.5. TRAIL

Tumor necrosis factor related apoptosis-inducing ligand (TRAIL/Apo2L) is a part of the TNF family and can induce programmed cell death of neoplastic cells while causing no toxicity against normal body tissues. TRAIL mediated apoptosis occurs following its binding to death receptors, TRAIL-R1 (DR4) and/or TRAIL-R2 (DR5). Mapatumumab is a humanized mAb that targets and triggers TRAILR1 receptor. The combination of mapatumumab with bortezomib showed promising results in preclinical studies that led to clinical studies of mapatumumab [68]. Belch et al. [69] studied mapatumumab in combination with bortezomib. They divided patients into three arms: Arm A - bortezomib alone, Arm B10 − mapatumumab 10 mg/kg + bortezomib, Arm B20 − mapatumumab 20 mg/kg + bortezomib. They reported ORR of 51.4%, 30.3% and 52.8% while the median duration of response was 8.5, 9.3 and 7.6 months in arms A, B10 and B20, respectively. Although there was no significant toxicity, mapatumumab failed to achieve significant clinical improvement. These results do not favor further studies of mapatumumab + bortezomib in RRMM patients.

### 3.3. Immune Checkpoint Inhibitors

#### PD-1/PD-L1

Investigators saw PD-1 expression on the surface of T-cells and expression of its ligand PDL-1 on tumor cells. PD-1/PDL-1 interaction inhibits the proliferation of T-cells. PD-1 expression on plasma cells is increased in the presence of bone marrow stromal cells and is associated with advanced disease [70]. mAbs against PD-1/PD-L1 block the escape of tumor cells from the immune system by enhancing T-cell function [71]. Nivolumab, a mAb against PD-1, achieved CR and OR in only one patient out of 27 RRMM patients when it was used as monotherapy [72]. The use of nivolumab in combination with ipilimumab (CTLA4 mAb) in 7 RRMM who failed 5 prior lines of therapy failed to achieve ORR and had results similar to nivolumab monotherapy [73]. Ribrag et al. studied pembrolizumab in 30 RRMM patients with 57% SD as the best response [74]. Pembrolizumab in combination with Pd were studies by Badros et al. in 48 patients who had 3 previous lines of therapy. Among 27 response evaluable patients, 4 had a stringent complete response (sCR), 3 had VGPR and 14 PR with mPFS of 17.4 months [75]. Investigators studied Pidilizumab that is another mAb against PD-1 in combination with lenalidomide. Only one patient achieved PR and VGPR was achieved by 3 patients out of 12 RRMM patients [76].

There have been many concerns regarding the safety of combination of mAbs targeting PD-1/PD-L1 with IMiDs. This is supported by data from KEYNOTE-183 [74] (pembrolizumab with pomalidomide and dexamethasone) and KEYNOTE-185 ^95^ (pembrolizumab with lenalidomide) trials that reported increased mortality, increased incidence of grade 3-5 adverse events in patients treated with pembrolizumab as compared to control group.

### 3.4. Antibody Drug Conjugate (ADC)

#### 3.4.1. CD56

CD56 (neuronal cell adhesion molecule) is a cell surface glycoprotein that is particularly expressed on NK cells, neuronal cells and cytotoxic cells [77]. We generally do not see expression of CD56 on benign B-cells and it can be expressed on more than 70% of MM cells [78]. Lorvotuzumab is anti-CD56 mAb that is conjugated to a maytansinoid cytotoxic agent known as DM-1 [79]. Channan et al. evaluated lorvotuzumab mertansine (LM) monotherapy in 37 patients with 6 previous lines of therapy and those patients did not achieve an OR [78]. Berdeja et al. evaluated LM in addition to Rd in 44 patients with median 2 previous lines of therapy. An ORR of 58%, 1 sCR, CR each and 8 VGPR were reported in 32 response evaluable patients. Peripheral neuropathy was the most commonly found an adverse effect. Investigators felt that this toxicity could be explained by CD56 distribution on neuronal and NK cells [80].

#### 3.4.2. CD138

CD138 (syndecan-1) is a heparan-sulfate coated glycoprotein that facilitates MM cell adhesion and its loss from the cell surface may lead to MM migration and metastasis [81]. It also functions as a co-receptor for MM growth receptors [82]. Indatuximab ravtansine (IR, BT-062) is the antibody drug conjugate of anti-CD138 chimerized mAb and cytotoxic maytansinoid DM4.

The binding of BT-062 and CD138 leads to CD138 internalization and release of anti-microtubule agent cytotoxic DM4 that that leads to MM cell death. Heffner et al. studied IR in 29 patients with medians of 2 previous lines of therapy as monotherapy and reported OR in one patient [83]. Kelly et al. evaluated IR + Rd in 64 patients with medians of 3 previous lines of therapy and reported an OR of 78%. Later they did head to head trials of IR + Rd versus IR + Pd. They reported no statistically significant difference in OR (78% vs. 79%) [84].

### 3.5. Future Prospective Therapies

#### 3.5.1. Bi-Specific Antibodies (BiAb)

**Figure 2:**
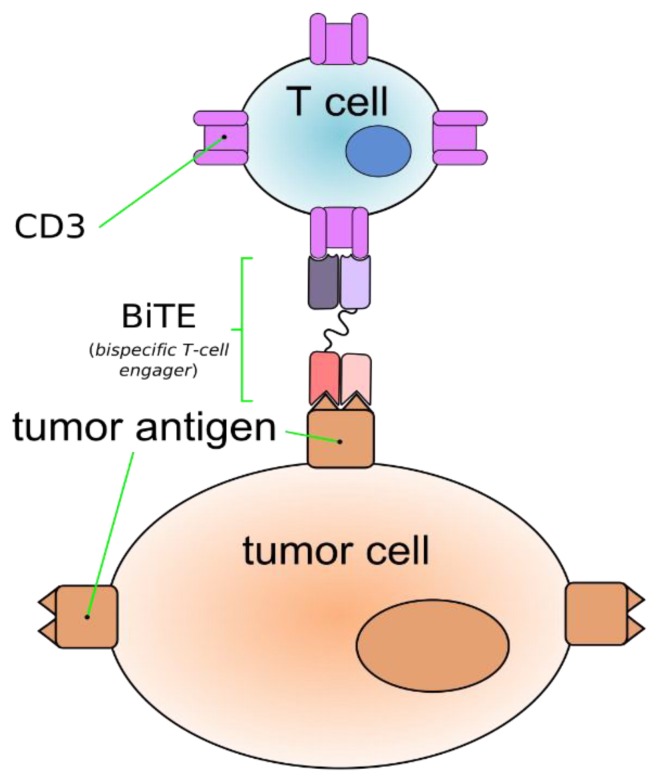
Bi-specific T-cell engaging antibody (BiTEs) bind tumor cells and immune cells through the interaction of CD3 with antigens on tumor cells.

Bi-specific antibodies are a novel approach in the treatment of MM. They have a unique potential to target two different antigens at the same time. These targets could be a cancer cell or these antibodies can target body`s immune cells and cancer cells at the same time. They involve both adoptive cytolytic and innate immune cells simultaneously against cancer cells. There are different kinds of bi-specific antibodies (BiAb) like Bi-specific T-cell engaging antibody (BiTEs) and bi-specific antigen binding fragments (BiFabs), depending on targets. BiTEs bind tumor cells and immune cells by means of the interaction of CD3 with antigens on tumor cells (Figure 2). This interaction activates T-cells that attack the targeted tumor cells [85]. Various BiTEs that target BCMA and CD3 such as EM801, JNJ-64007957 and BI 836909 have shown favorable results in in vivo and in vitro models of MM [86,87]. Topp et al. studied AMG 420 (previously BI 836909) in 35 RRMM patients with ≥2 prior lines of therapy. At dose of 400ug they reported ORR of 83% (5/6) with 3 patients having MRD-negative CRs.

Several clinical trials are currently ongoing to study BiTEs. PF-06863135 is an anti-CD3/anti-BCMA bispecific monoclonal antibody for MM patients (NCT03269136, NCT03145181, NCT03269136 and NCT02514239). There are a few other BiTEs in pre-clinical studies combining CD3 and CD138 that showed significant activity against MM in murine and in vitro models [88]. NKG2D is an activation receptor present on CD8^+^ T-cells, NK cells, γδ T-cells and NKT-cells. NKG2D and CS1 tumor related antigens represent a possible target by bi-specific antibodies. A bi-specific antibody targeting NKG2D and CS1 simultaneously has shown an enhanced killing of MM cells in vivo models [89]. There is another novel approach to treat MM using BiFabs that are bi-specific antibodies which target two distinct epitopes on the same antigen. Comparison of BiFabs targeting BCMA and CS1 demonstrated that BiFab-BCMA had greater anti-myeloma potential than BiFab-CS1. BiFab-BCMA specifically directs T-cells for MM cell lysis and produces results similar to CAR-T BCMA in vivo and in vitro studies [90]. Cytokine release syndrome is a form of cytokine storm that can happen as a dangerous complication of extreme T-cell activation.

#### 3.5.2. CD47

TTI-622 is recombinant soluble fusion protein that is produced by linking sequences coding N-terminal CD47 binding domain of human SIRPα with Fc domain of IG4. TTI-622 binds human CD47 and prevents the delivery of inhibitory “do not eat” signal to macrophages. There is an ongoing phase 1a/1b trial of TTI-622 in the treatment of advanced relapsed or refractory multiple myeloma and lymphoma [91].

## 4. Discussion

An extensive review of the literature showed that isatuximab (anti-CD38) and F50067 (anti-CXCR4) were the only two mAbs that produced encouraging results as monotherapy with ORR of 66.7% and 32% respectively [32,92]. Isatuximab used in combination with Rd produced CBR of 83% and in combination with Pd produced CBR of 73% [93,94]. However, the clinical trial of F50067 with both monotherapy and combination of Rd was discontinued due to significant hematological toxicities [32]. Our review also shows that trials using indatuximab, pembrolizumab, lorvotuzumab, siltuximab and dacetuzumab in combination therapy produced better outcomes as compared to monotherapies in RRMM.

Immune therapy has been of increasing interest as the immune system is significantly impaired with negative effects on both humoral and cellular immunity [16]. Antibody drug conjugates are an emerging treatment modality for MM because of their targeted nature. Indatuximab ravtansine could not produce favorable outcomes as a monotherapy [68] but produced an ORR 78% and 79% in combination with Rd and Pd, respectively [69,70]. Similarly, Lorvotuzumab mertansine did not achieve an ORR as a monotherapy but achieved an ORR of 59% in combination with Rd [64,65]. These results highlight the possibility of combination therapies being effective where original monotherapy was not. Another possible treatment strategy of immune checkpoint inhibitors couldn`t show a beneficial effect in RRMM and was associated with prohibitive toxicities [70,95,96,97].

Investigators seeking future treatment recommendations should seek guidance from data produced in large well-designed randomized phase II-III trials, but they can formulate preliminary predictions. Due to the wavering escape strategies of myeloma cells, the role of precision medicine utilizing immunotherapies as a cornerstone in combination with approved therapies to treat MM is evolving [98]. It is challenging to target multiple evolving abnormalities at a precise time, in the right sequence and with the correct combination to prevent future relapses. This forms the basis of on-going trials evaluating 4 drug treatment strategies with or without a mAb compared to standard of care triple drug regimens [14].

Many questions remain unanswered, most relevant to immune therapy being the ideal timing of mAb in clinical course and the role of genomic profiling. It seems logical to use targeted immuno-modulation early in the disease when the immune system is more capable of responding but toxicities may be offset some of these approaches. Patient who cannot achieve consolidation with high dose melphalan and autologous transplant may benefit from earlier use of mAbs. Understanding and controlling the adverse effects associated with these drugs will also be important.

Despite advances in understanding, different options are deployed in a relatively empiric fashion, relying on disease characteristics clinically rather on genotype, molecular features or mutations [99]. Receptor density studies [100], immuno-phenotyping and emerging understanding about molecular minimal residual disease (MRD) status may be of utility in deciding drugs for induction and maintenance therapy [101,102] and aid in determining when to stop any drug treatment altogether based on MRD negativity in order to ultimately reduce the financial burden and clinical toxicity [15,103]. With further treatment insight about biological drivers, checkpoint inhibitors, tumor microenvironment and interaction with the tumor cells we can hope to approach the threshold of treatment by individualizing regimen for each patient.

## Figures and Tables

**Table 1:** Surface receptor targeting antibodies in relapsed refractory multiple myeloma.

Author, Year, Study Design, Journal.	No of Patients	Antibody	Target	Regimen	Median Prior Therapies	Dose	No. of Cycles	Clinical Outcome
Agura, 2009, Phase 1b, Blood.	36/33 (REP)	Dacetuzumab (IgG1)	CD-40	Dac + Len + Dex	4	4–12 mg/kg	4	OR = 39%, CR = 3%, PR = 33%, MR = 12%, SD = 30%, PD = 6%, NE = 12%
Husein, 2010. Phase I, Haematologica.	44	Dacetuzumab (IgG1)	CD-40	Dacetuzumab Monotherapy	5 (2–14)	4–12 mg/kg	4–5	SD = 20%
Bensinger, 2012, Phase I, British Journal of Haematology.	28	Lucatumumab (IgG1)	CD-40	Lucatumumab Monotherapy	NR	1–6 mg/kg	4	SD = 43%, PR = 4% > 8 m
Martin, 2014, Phase I, ASH.	35	Isatuximab (SAR650984) (IgG1-kappa)	CD-38	Isatuximab Monotherapy	6 (2–14)	0.1–20mg/kg	5–7	ORR = 32%(>10mg), PR = 6, CR = 2
Richter, 2016, Phase II, ASCO.	97	Isatuximab SAR650984 (IgG1-kappa)	CD-38	Isatuximab Monotherapy	5 (2–14)	3–10 mg/kg	NR	ORR = 24% at dose > 10 mg/kg
Martin, 2014, Phase Ib, ASH.	31	Isatuximab SAR650984 (IgG1-kappa)	CD-38	SAR + Len + Dex	6 (2–12)	3, 5, 10 mg/kg	NR	ORR = 64.5%, CBR = 70.8%, sCR = 6%, VGPR = 26%, PR = 32%, PFS = 6.2 m
Martin, Phase Ib, 2017, Blood.	57	Isatuximab (IgG1-kappa)	CD-38	Isatuximab + Len + Dex	5 [1–12]	3, 5 or 10 mg/kg [Q2W] or 10 or 20 mg/kg weekly	9 (1–37)	ORR = 56% (29/52), mPFS 8.5 months
Lendvai, 2016, Phase Ib, Haematologica	26	Isatuximab (IgG1-kappa)	CD-38	Isatuximab+ Lenalidomide+ Dexa	4.5(1–8)	10 mg/kg+ 25 mg+40 mg	NR	ORR: 50%, VGPR: 25%, PR: 25% CBR (m > MR): 83%
		Isatuximab (IgG1-kappa)	CD-38	Isatuximab + Lenalidomide + Dexa	6(3–10)	20mg/kg + 25mg + 40mg	NR	ORR: 50%, VGPR: 20%, PR: 30% CBR (m > MR): 50%
Mikhael, 2017, Phase Ib, Haematologica.	26	Isatuximab (IgG1-kappa)	CD-38	Isatuximab + Pomalidomide + Dexa	4 (2–11)	5, 10, 20 mg/kg+ 4 mg+ 40 mg	NR	CBR: 73%, PR: 62% (*n* = 16) {CR = 1; VGPR = 8, PR = 7}
Raab, 2016, Phase I/IIa, Blood.	16 REP	MOR202 (IgG λ)	CD-38	MOR202 monotherapy	4	4, 8 and 16 mg/kg weekly.	NR	PR 19%, VGPR 13%
	7/5 (REP)	MOR202 (IgG λ)	CD-38	MOR202 + LEN cohort	4	4, 8 and 16 mg/kg weekly.	NR	PR 71%
	5/3 (REP)	MOR202 IgG λ)	CD-38	MOR202 + POM	4	4, 8 and 16 mg/kg weekly.	NR	CR = 2
Benson, 2015, phase I, Clinical Cancer Research.	15	IPH 2101 (IgG4)	KIR	IPH 2101 + Len (10-25mg)	1–2	0.2–2 mg/kg	4	VGPR 13%, PR 20%, MR 7%, SD 40%, PD 20%
Benson, 2012, Phase I, Blood.	32	IPH 2101 (IgG4)	KIR	IPH 2101 Monotherapy	2 (1–7)	0.0003–3 mg/kg every 28 days	4	No ORR, SD *n* = 11 (34%)
Kaufman, 2013, Phase I, British Journal of Haematology.	25	Milatuzumab (IgG1-kappa)	CD74	IPH 2101 Monotherapy	5	1.5–16 mg/kg × 2 or 4 weeks	8	No ORR, SD = 26% (5/19) >3 m, (1/19)>17 m
Hansson, 2015, Phase I, Clinical Cancer Research.	35/29 (REP)	BI-505 (IgG1)	ICAM-1	BI-505 Monotherapy	6	0.0004 to 20 mg/kg	1–2	SD = 24% (2 m), PD = 65%
Lacy, 2008, Phase I, Journal of Clinical Oncology.	47	Figitumumab (CP 751,871) IgG2	IGF-1	Figitumumab + Dex if no PR on figitumumab monotherapy	4 (0–8)	0.025–20 mg/kg for 4 weeks	4	No objective response
	27	Figitumumab (CP 751,871) IgG2	IGF-1	Figitumumab + Dex	4 (0–8)	0.025–20 mg/kg for 4 weeks + 40 mg /day Dex		PR = 6
Moreau, 2011, Phase I, Leukemia.	15	AVE1642 (IgG1)	IGF-1	AVE1642 monotherapy	4	3–18 mg/kg	2	MR = 1, SD = 7, PD = 4
	11	AVE1642 (IgG1)	IGF-1	AVE1642 + Bortezomib	4	0.5–12 mg/kg + 1.3 mg/m^2^	4	CR = 1, PR = 1, SD = 3
Rasche, 2015, Phase I, Haematologica.	12	PAT-SM6 (IgM)	GRP-78	PAT-SM6	3.9 (2–7)	1,3,6 mg/kg/day	4	No OR, SD: 33.3%

Abbreviations: REP; Response evaluable patients, Len; Lenalidomide, Dex; Dexamethasone, KIR; Killer-cell immunoglobulin like receptor, ORR; Objective response rate, CR; Complete response, PR: partial response, VGPR; Very good partial response, MR; Minimal response, PD; Progressive disease, NE; Not evaluable, PFS; progression free survival, m; months. GRP: Glucose regulated protein, ASH; American Society of Hematology, ASCO; American Society of Clinical Oncology.

**Table 2:** Non-surface receptor Antibodies targeting in Relapsed Refractory Multiple Myeloma.

Author, Year, Study Design	No of Patients	Antibody	Target	Median Prior Therapies	Dose	No. of Cycles	Regimen	Outcome
Callander, 2009, NEJM.	31/27(REP)	Bevacizumab (IgG1-kappa)	VEGF	3 (1–7)	Bevacizumab 10 mg/kg × 2 weeks	4	Bevacizumab + Len (25mg) + Dex (40mg)	OR = 70%, CR = 15%, PR = 56%, PD = 11%
Somlo, 2011, Phase II, British Journal of Haematology.	6	Bevacizumab (IgG1-kappa)	VEGF-A	3 (0–5)	Bevacizumab 10 mg/kg	4	Bevacizumab Monotherapy	PD = 29–69 days, SD = 238 days, SD = 16.6%, PD = 83%
	6	Bevacizumab (IgG1-kappa) ± Thalidomide	VEGF-A			4	Bevacizumab ± Thalidomide	SD = 37–350 days, PR = 33%, PD = 67%
White, 2013, Phase II, Cancer.	49	Bevacizumab (IgG1-kappa)	VEGF	(1–3)	Bevacizumab 15 mg/kg I.V	8	Bevacizumab + Bor	ORR = 51%, PR = 16.3%, mPFS = 6.2 m
	53	Placebo			Bor 1.3 mg/m^2^		Placebo + Bor	ORR = 43.4%, PR = 7.5%, mPFS = 5.1 m
Brighton, 2017, Phase II, ASH.	74	Siltuximab (IgG1)	IL-6	NR	15 mg/kg Q4 week vs. Placebo	NR	Siltuximab vs. placebo	1-yr PFS 84.5% with siltuximab vs. 74.4% with placebo.
Orlowski, 2015, Phase II, American Journal of Hematology.	142	Siltuximab (IgG1)	IL-6	1–3	Siltuximab 6 mg/kg	4	Siltuximab + Bor	mPFS = 8m, ORR = 55%, CR = 11%, OS = 30.8 m
	139	Placebo			Placebo		Placebo + Bor	mPFS = 7.6, ORR = 47%, CR = 7%, OS = 36.8 m
Voorhesse, 2009, Phase II, British Journal of Haematology.	14	Siltuximab (IgG1)	IL-6	4	6 mg/kg	4	Siltuximab monotherapy	No Response (CR/PR), SD = 62%, PD = 39%
	39	Siltuximab (IgG1)			6 mg/kg + 40g		Siltuximab + Dex	ORR = 23%, PR = 17%, MR = 6%, SD = 57%, PD = 17%, PFS = 3.7 m
Suzuki, 2015, Phase I, International Journal of Hematology.	9	Siltuximab (IgG1)	IL-6	1–2	5.5/11 mg/kg	≥ 9	Siltuximab + Bor (1.3 mg/m^2^) + Dex (20 mg)	CR = 22%, PR = 44%
Rossi,2009, Phase I, British Journal of Cancer.	12/11(REP)	Atacicept (IgG)	BAFF	NR	2–10 mg/kg	5	Atacicept monotherapy	No ORR, PD = 54%, SD = 45%
Lida, 2016, Phase I, Cancer Science.	4	Tabalumab (IgG4)	BAFF	At least 1	100 mg + 1.3 mg/m^2^ + 20 mg	3(2–11)	Tabalumab + Bor+ Dexa	ORR:100%, VGPR: 50% (*n* = 2), PR: 50% (*n* = 2)
	12	Tabalumab (IgG4)	BAFF	At least 1	200 mg + 1.3 mg/m^2^ + 20 mg	4.5 (1–15)	Tabalumab + Bor + Dexa	ORR: 41.7%, VGPR: 8.3% (*n* = 1), PR:33.3 %(*n* = 4), SD:16,7 %(*n* = 2), PD:25%(*n* = 3)
Reje, 2017, Phase II, British Journal of Haematology.	74	Tabalumab (IgG4)	BAFF	1–3	100 mg	8 or 10	Tab + Bor + Dex	ORR = 58.1%
	74	Tabalumab (IgG4)	BAFF		300 mg		Tab + Bor + Dex	ORR = 59.5%
	72	Placebo			no mAb		Placebo + Bor + Dex	ORR = 61.6%
Lesokhin, 2016, Phase Ib, JCO.	27	Nivolumab (IgG4)	PD-1	3 (1–12)	1–3 mg/kg x 2wk	NR	Nivolumab monotherapy	mPFS = 10 wk=K8, OR = 4%, SD = 63%, CR = 4%
Ansell, 2016, Phase I, ASH.	7	Nivolumab (IgG4) + Ipilimumab (IgG1)	PD-1 + CTLA-4	5 (range 2–20)	3 mg/kg IV and 1 mg/kg IV every 3 weeks × 4 followed by Nivo 3 mg/kg every 2 week for up to 2 years.	NR	Nivolumab + Ipilimumab	mPFS = 2.2, mOS = 7.6, No ORR. SD 1 (14%)
Badros, 2017, Phase II, Blood.	48	Pembrolizumab (IgG4)	PD-1	3 (2–5)	200 mg IV × 2 wk	28	Pembrolizumab + pom + Dex	27 of 48 pts (56%) ORR > PR; sCR (n = 4, 8%), nCR (*n* = 3, 6%), VGFR (*n* = 6, 13%), PR (*n* = 14, 29%).
Ribrag, 2017, Phase Ib, Haematologica.	30	Pembrolizumab (IgG4)	PD-L1	4(2–12)	100–200 mg/kg Qweek or Q 2week.	6 (2–15)	Pembrolizumab monotherapy	SD: 57%. PD: 43%.
Efebera, 2015, Phase I/II, Blood.	12	Pidilizumab (IgG4)	PD-1	2 (2–11)	1.5–6 mg/kg every 28 days	NR	Pidilizumab + Len (15–25mg)	VGPR *n* = 3, PR *n* = 1
Fouquet, 2018, Phase I, Oncotarget.	10/6 (REP)	F50067 (IgG1)	CXCR4	NR	Dose-Group (mg/kg)s were analyzed for MDD 0.03, 0.1, 0.3, 1.0	21	F50067 Monotherapy	ORR 66.7% (>PR). Objective response 66.7% (>SD)
	4/3 (REP)	F50067 (IgG1)	CXCR4	NR	0.03, 0.1. weekly or Q2 week	15	F50067 + Len-LoDex	Objective response 33.3% (>SD) ORR not available
Belch, 2011, Phase II, Haematologica.	Arm A = 35	No mAb, only Bor	TRAILR1	1.6	Velcade Dose: 1.3 mg/m^2^ on days 1, 4, 8, 11 Q21 D	Maximum 17 cycles (1year)	Bor	ORR 51.4% Median DOR 8.5 m. PR 18. Mean PFS 8.7 CI(7.6, 10.0)
	Arm B10 = 33	Mapatumumab (IgG1)	TRAILR1	1.6	10 mg/kg on d1 Q21 days	Maximum of 17 cycles (1year)	Bor + Mapatumumab	ORR 30.3%, Median DOR 9.3 m. PR 10. Mean PFS 4.7 CI(2.5, 7.4) *p* = 0.29
	Arm B20 = 36	Mapatumumab (IgG1)	TRAILR1	1.6	20 mg/kg on Day 1 Q21 days	Maximum of 17 cycles (1year)	Bor + Mapatumumab	ORR 52.8%, Median DOR 7.6 m. PR 17 Mean PFS 5.7 CI(5.2, 8.9) *p* = 0.21
Channan, 2010, Phase I, Blood.	37	Lorvotuzumab mertansine (ADC) (IgG1)	CD56	6	40–140 mg/m^2^ × wk	NR	Lorvotuzumab mertansine Monotherapy	SD = 41%
Berdeja. 2012, Phase I, JCO.	44 (39REP)	Lorvotuzumab mertansine (IgG1)	CD56	2 (1–11)	75–112 mg/m^2^	NR	LM + LEN (20mg) + Dex (40mg)	ORR = 59%, sCR *n* = 1, CR *n* = 1, VGPR *n* = 8, PR *n* = 9
Heffner, 2012, Phase I/IIa, Blood.	29/23 (REP)	Indatuximab Ravtansine (ADC) (IgG1)	CD138	2 (1–11)	40–160 mg/m^2^	NR	Indatuximab Monotherapy	PR = 1, SD = 11, mPFS = 112 days (90–245)
Kelly K. R., 2014, PhaseI/IIa, Blood.	45/36 (REP)	Indatuximab Ravtansine (ADC) (IgG1)	CD138	3	80, 100, 120 mg/m^2^	NR	Indatuximab + dex + Len	ORR = 78%, sCR = 1, CR = 2, VGPR = 10, PR = 15, SD = 2
Kelly K. R., 2016, PhaseI/IIa, Blood.	47/43 (REP)	Indatuximab Ravtansine (ADC) (IgG1)	CD138	1–6	80–100 mg/m^2^	NR	Indatuximab + dex + Len	ORR = 78%, PR = 33/47, mPFS 16.4m
	17			> 2		NR	Indatuximab + dex + Pomalidomide	ORR = 79%, VGPR = 4, PR = 7

Abbreviations: REP; Response evaluable patients, mAb; Monoclonal Antibodies, ADC; Antibody Drug Conjugate, BAFF; B cell activating factor, len; Lenalidomide, Dex; dexamethasone, Bor, Bortezomib, Tab; Tabalumab, pom; Pomalidomide, pem; pembrolizumab, m; months, ORR; Objective response rate, CR; Complete response, PR: partial response, VGPR; Very good partial response, MR; Minimal response, PD; Progressive disease, NE; Not evaluable, mPFS; median Progression free survival, sCR; stringent complete response, SD; Stable disease, NR; Not reported, wk; weeks, LM; Lorvotuzumab mertansine, NEJM; New England Journal of Medicine, ASH; American Society of Hematology, JCO; Journal of Clinical Oncology.

**Table 3:** Adverse effects of monoclonal Antibodies drugs.

Author, Year, Study Design	Antibody	Adverse Effects ≥ Grade III	Common Adverse Effects
Hansson,2015, Phase I	BI-505	Headache (*n* = 4), Pyrexia (*n* = 3), Infusion related reactions (*n* = 1), Fluid overload (*n* = 1), T-wave inversion (*n* = 1).	Fatigue (47%), Pyrexia (32%), Headache (32%), Nausea (29%), Chills (24%)
Callander, 2009	Bevacizumab	DVT (*n* = 3), SOB (*n* = 2), A fib (*n* = 3)	Fatigue
Somlo, 2011, Phase II	Bevacizumab	Fatigue (16.6%), HTN (16.6%), Neutropenia (16.6%), Hyponatremia (16.6%)	NR
	Bevacizumab ± Thalidomide	Lymphopenia (16.6%), Fatigue (16.6%), Pulmonary HTN (16.6%)	
White, 2013, Phase II	Bevacizumab + Bortezomib	Thrombocytopenia (28%), Neutropenia (18%)	Anemia, Diarrhea, Fatigue, URTI, Neuralgia
	Bortezomib + Placebo	Thrombocytopenia (30%), Diarrhea (10%)	Anemia, Diarrhea, Fatigue, URTI, Neuralgia
Rasche, 2015, Phase I	PAT-SM6	Neutropenia (8.3), Back pain (8.3), bile duct stone (8.3)	Neutropenia (50), Leukopenia (67)
Orlowski, 2015, Phase II	Siltuximab + Bortezomib	Neutropenia (49%), Thrombocytopenia (48%)	Infections (62%), Sensory neuropathy (49%)
	Bortezomib + Placebo	Neutropenia (24%), Thrombocytopenia (34%)	Infections (49%), Sensory neuropathy (51%)
Agura, 2009, Phase 1b	Dacetuzumab	Herpes Zoster, Renal failure	Infusion reactions, grade (I/II), Fatigue (47%), Neutropenia (28%), Thrombocytopenia (25%), Diarrhea (22%), Constipation (19%), Headache (19%)
Husein, 2010, Phase I	Dacetuzumab	Total grade 3 AE = 30 %, Thrombocytopenia (7%), Aseptic meningitis (5%), Renal failure (5%)	Fatigue (57%), headache (43%), nausea (23%), anemia (21%). Elevated LFTs (41%), anorexia, back pain, constipation, diarrhea, ocular hyperemia (21%)
Bensinger, 2012, Phase I	Lucatumumab	Thrombocytopenia (4%), Increased LFTs (4%), Increased Lipase (4%)	Infusion reactions, Anemia (7%), Hypercalcemia (7%), Pyrexia (7%)
Martin, 2014, Phase I	Isatuximab (SAR650984)	Pneumonia, Fever, Hyperglycemia, Hypophosphatemia,	Pneumonia 9%, fever (3%), apnea (3%), fatigue (3%), hyperglycemia (3%)
Richter, 2016, Phase II	Isatuximab SAR650984	NR	Nausea (33%), Fatigue (30%), Dyspnea (26%), Infusion related 49%
Martin. Phase Ib, 2017	Isatuximab + Len + Dex	pneumonia (9%), fatigue (7%). Hypokalemia, anaphylaxis, febrile neutropenia (5% each).	Thrombocytopenia (38%), anemia (25%), Neutropenia (60%),
Martin, 2014, Phase Ib	Isatuximab + Len + Dex	No DLT reported, IAR (6%)	Fatigue (41.9%), Nausea (38.7), URTI(38.7%), Diarrhea(35.5%)
Voorhesse, 2009, Phase II	Siltuximab	Thrombocytopenia, anemia, Neutropenia, abnormal LFTs, fatigue.	Diarrhea (29%), Nausea (22%), Constipation (20%), Fatigue (43%) Peripheral edema (29%)
	Siltuximab + Dexamethasone		
Suzuki, 2015, Phase I	Siltuximab	No DLT, Lymphopenia (89%), Neutropenia (44%)	Abnormal LFTs (44%), Rash (44%), Hyperlipidemia (44%)
Rossi,2009, Phase I	Atacicept	Neuropathy, Epiploic appendicitis.	Infections, Bone Pains
Shinsuke Iida, 2016, Phase I	Tabalumab	Febrile Neutropenia, Tumor lysis syndrome, Ileus.	Thrombocytopenia (81.3%), Lymphopenia (43.8%), Increased alanine aminotransferase (43.8%)
Reje, 2017, Phase II	Tabalumab	Thrombocytopenia (12 8%), Pneumonia (9.1%)	Thrombocytopenia (37%), Fatigue (37%), Diarrhea (35%), Constipation (32%)
	Placebo		
Lesokhin, 2016, Phase Ib	Nivolumab	Pneumonitis (4%), Myositis (4%), Raised CPK (4%)	seen in 52% patients
Badros, 2017, Phase II	Pembrolizumab	Hematologic (40%), Hyperglycemia (25%), pneumonia (15%)	Pancytopenia (13%), Hypothyroidism (10%)
Efebera, 2015, Phase I/II	Pidilizumab	Anemia 25%, neutropenia 23%, thrombocytopenia 34%	Fatigue (50%), anorexia (17%), hypophosphatemia (17%)
Channan, 2010, Phase I	Lorvotuzumab mertansine	Peripheral neuropathy, Fatigue, Acute renal failure	Fatigue, peripheral neuropathy, Headache, Raised AST
Berdeja, 2012, Phase I	Lorvotuzumab mertansine	Peripheral neuropathy, Neutropenia *n* = 1, Hyperuricemia Tumor lysis syndrome *n* = 2	Peripheral neuropathy (42%)
Heffner, 2012, Phase I/IIa	Indatuximab Ravtansine (ADC)	Palmar-planter eryhtrodysesthesia syndrome (*N* = 1), Elevated LFTs	Fatigue, Anemia, Diarrhea
Kelly, 2014, PhaseI/IIa	Indatuximab Ravtansine (ADC)	Mucosal inflammation (*n* = 1), Anemia (*n* = 1)	Fatigue, Hypokalemia, Diarrhea
Kelly, 2016, PhaseI/IIa	Indatuximab Ravtansine (ADC)	NR	Diarrhea, Fatigue, Nausea
Benson, 2015, phase I	IPH 2101	leucopenia *n* = 1, neutropenia *n* = 1	Myelodysplasia *n* = 1, neutropenia, IRR
Benson, 2012, Phase I	IPH 2101	NR	Fatigue *n* = 10, Chills *n* = 5, pyrexia *n* = 5
Kaufman,2013, Phase I	Milatuzumab	Anemia 20%, CRS 4%, Hypokalemia 4%, Epistaxis 4%	Nausea (48%), Fever (36%), CRS (20%), Headache (20%), HTN (20%)
Lacy, 2008, Phase I	Figitumumab (CP 751,871)	Anemia (2.1%), Hyperglycemia (2.1%)	Anemia (6.4%), Increased AST (6.4%)
Lacy, 2008, Phase I	Figitumumab (CP 751,871)	Muscle weakness (3.7%), Increased AST (3.7%)	Anemia (7.4%), Increased ALT (11%)
Moreau,2011, Phase I	AVE1642	Grade III hyperglycemia *n* = 1	NR
Moreau,2011, Phase I	AVE1642	Hypercalcemia *n* = 1, renal vein thrombosis *n* = 1	NR
Miguel, 2014, Phase II	VMP + Placebo	All = 81%, Neutropenia (43%), Thrombocytopenia (25%), Pneumonia (17%), Median PFS = 17m	Infections (17%), GI disorders (11%)
Miguel, 2014, Phase II	Siltuximab + VMP	All = 92%, Neutropenia (62%), Thrombocytopenia (44%), Pneumonia (17%), Median PFS = 17m	Infections (29%), GI disorders (11.5%)
	Siltuximab	Pneumonia, Thrombocytopenia	Fatigue (63.6%), Constipation (54.5%), Paresthesia (45.5%), Myalgia (56.4%)
Baz, 2007, Phase II	Rituximab + MP	Diarrhea (31%), Neutropenia (51%), Anemia (47%), Thrombocytopenia (40%)	Fever, fatigue, cough, dyspnea, diarrhea, nausea, diarrhea and constipation. Possible AE related to rituximab were IRR (11%)
Vorhees, 2013, Phase II	Siltuximab	Thrombocytopenia 28%, Anemia 43%, Neutropenia 7%	Neutropenia 29%, Anemia 35%, Thrombocytopenia 49%, Fatigue 43%, Abnormal Hepatic Function 31%, Diarrhea 29%, Edema 29%, Dyspnea 27%, Dizziness 25%, Nausea 28%, Insomnia 28%, Weight increase 20%
	Siltuximab + Dexa	Thrombocytopenia 26%, Anemia 16%, Neutropenia 18%, Fatigue 8%	NR
Ribrag, 2017, Phase I	Pembrolizumab	Myalgia 3%	Asthenia 17%, Pruritus 3%, Arthralgia 3%, Fatigue 3%, Hyperglycemia 3%, Blurred vision 3%, Aspartate Aminotransferase increased 3%
Rasche, 2015, Phase I	PAT-SM6	Neutropenia 8%	Neutropenia 50%, Leukopenia 66%, Increase in C reactive protein 8%, Hypertriglyceridemia 8%
Raje, 2016, Phase I	Tabalumab	Peripheral Sensory Neuropathy 15%, Fatigue 6%, Diarrhea 8%m Thrombocytopenia 31%m Anemia 6%, Neutropenia 15%, Pneumonia 13%, Hypokalemia 8%, Renal Failure 8%, Gi Hemorrhage 4%, Musculoskeletal pain 6%	Peripheral Sensory Neuropathy 63%, Fatigue 58%m Diarrhea 54%, Nausea 48%, Thrombocytopenia 33%, Anemia 23%,
Raab, 2016, Phase I/IIa	MOR 202	NR	IRRs 10%
Patnaik, 2014, Phase I	Ficlatuzumab	Hyper/Hypokalemia, Diarrhea, Fatigue	Peripheral edema, fatigue, nausea
Orlowski, 2015, Phase II	Siltuximab + placebo	AE grade >3: 74%. Neutropenia 29%, Thrombocytopenia 34%, Bleeding events <2%, Infections 14%	Neutropenia 36%, Thrombocytopenia 45%, Peripheral Sensory Neuropathy 51%, Diarrhea 35%, Anemia 29%, Fatigue 27%, Infection 49%
	Siltuximab + Bortezomib	AE grade >3: 91%. Neutropenia 49%, Thrombocytopenia 48%, Bleeding events <2%, infection 16%	Neutropenia 59%, Thrombocytopenia 57%, Peripheral Sensory Neuropathy 49%, Diarrhea 35 = 6%, Anemia 31%, Fatigue 27%, Infection 62%
Mikhael, 2017, Phase IB	Isatuximab + Dexa Pomalidomide	Neutropenia 92%, Thrombocytopenia 32%	Fatigue 62%, Diarrhea 35%, Dyspnea 31%
Martin, 2017, Phase IB	Isatuximab + Lenalidomide + Dexa	Anemia 25%, Lymphopenia 58%, Neutropenia 60%, Leukopenia 53%, Thrombocytopenia 38%, Fatigue 7%, Pneumonia 9%, Febrile Neutropenia 5%, Anaphylactic Reaction 5%, Hypokalemia 5%	Anemia 98%, Lymphopenia 95%, Neutropenia 89%, Leukopenia 91%, Thrombocytopenia 91%, IARs 56%, Diarrhea 53%, Fatigue 49%, URTI 40%, Nausea 35%, Insomnia 32%, Cough 26%, Headache 23%, Muscle spasm 23%, Vomiting 23%
Lida, 2016, Phase I	Tabalumab + Bortezomib + Dexa	>Grade3 AE: 81.3%	Thrombocytopenia 81%, Lymphopenia 43%, Anemia 31%, Increase Alanine Aminotransferase 43%, GI disturbances 62%, Constipation 38%
Lendvai, 2016, Phase Ib	Isatuximab + Dexa Lenalidomide	NR	IARs 65%, Fatigue 46%, Pyrexia 35%, Diarrhea 31%
Fouquet, 2018, Phase I	F50067 + Dexa Lenalidomide	Thrombocytopenia 64%, Neutropenia 57%, Anemia 14%	Thrombocytopenia 100%, Neutropenia 93%, Asthenia 7%, Hyperhidrosis 7%, Pyrexia 7%, Dyspnea 7%, Pulmonary Embolism 7%, Femoral Neck Fracture 7%, Rectal hemorrhage 7%
Brighton, 2017, Phase II	Siltuximab	NR	Infections and Infestations 38%, Renal and Urinary Disorders 7%
	Placebo	NR	Infections and Infestations 33%, Renal and Urinary Disorders 16%
Belch, 2011, Phase II	Bortezomib +/- Mapatumumab	Overall >Grade 3 AE. Arm A: 88.6%, Arm B10: 69.7%, Arm B20: 61%	Hematological, Peripheral Sensory Neuropathy
Badros, 2016	Pembrolizumab + Pomalidomide + Dexa	Anemia 21%, Neutropenia 40%, Lymphopenia 15%, Thrombocytopenia 8%, Fatigue 15%, Hyperglycemia 25%, URI 25%, Rash 10%	Dyspnea 54%, Dizziness 44%, Increased Creatinine 38%, Edema 35%, Rash 30%, Interstitial Pneumonitis 13%, Hypothyroidism 10%
Ansell, 2016, Phase I	Nivolumab + Ipilimumab	NR	NR

Abbreviations: ADC; Antibody drug conjugate, VMP; Bortezomib melphalan prednisone, MP; Melphalan prednisone, GI; Gastrointestinal, CRS: Complement release syndrome, IRR; Infusion related reactions, DLT; Dose limiting toxicity, PFS: Progression free survival, NR: not reported, Dexa; dexamethasone.
